# A concept analysis of dignity-protective continence care for care dependent older people in long-term care settings

**DOI:** 10.1186/s12877-020-01673-x

**Published:** 2020-07-29

**Authors:** Joan Ostaszkiewicz, Virginia Dickson-Swift, Alison Hutchinson, Adrian Wagg

**Affiliations:** 1 Centre for Quality and Patient Safety Research – Barwon Health Partnership, Institute for Healthcare Transformation, Deakin University, Geelong, VIC 3220 Australia; 2 School of Nursing and Midwifery, Deakin University, Gheringhap St, Geelong, VIC 3220 Australia; 3 National Ageing Research Institute, P.O Box 2127, Royal Melbourne Hospital, 21, Melbourne, VIC 3530 Australia; 4 Centre for Quality and Patient Safety Research – Monash Health Partnership, Institute for Healthcare Transformation, Deakin University, Burwood, VIC 3125 Australia; 5 Department of Medicine, Faculty of Medicine and Dentistry, University of Alberta, Edmonton, Alberta Canada

**Keywords:** Dignity, Continence care, Concept analysis, Person-centred care, Dignity-protective continence care, Long-term care

## Abstract

**Background:**

Although codes of conduct, guidelines and standards call for healthcare practitioners to protect patients’ dignity, there are widespread concerns about a lack of attention to the dignity of older people who need assistance with toileting, incontinence or bladder or bowel care in health or social care settings that provide long-term care. Incontinence and care dependence threatens patient dignity. The aim of this research was to explore, describe and explain the concept of dignity as it relates to continence care for older people requiring long-term care.

**Methods:**

The first four steps of Rodgers evolutionary method of concept analysis were followed. First, a comprehensive and systematic search of databases and key guidelines about continence care was undertaken to identify empirical research about dignity and continence care in older people in facilities that provide permanent residential or inpatient care of older people for day-to-day living. Data were extracted on the authors, date, sample, country of origin, and key definitions, attributes, contexts and consequences from each included record. Findings were inductively analysed and grouped according to whether they were the key attributes and antecedents of dignity in relation to continence care or the consequences of undignified continence care.

**Results:**

Of 625 articles identified, 18 were included in the final analysis. Fifty individual attributes were identified that were categorised in 6 domains (respect, empathy, trust, privacy, autonomy and communication). A further 15 were identified that related to the environment (6 physical and 9 social). Key consequences of undignified continence care were also identified and categorised into 3 levels of impact (resident/family member, staff or organisation).

**Conclusions:**

This research resulted in a conceptual understanding of dignity that can be used as a value or guiding principle in an ethic of care for older people who need assistance with toileting, incontinence or bladder or bowel care in long-term care settings.

## Background

The importance of dignity as core concept in health care is widely recognised. Dignity is a key aspect of patient care [1–5] that has been explored across a range of care disciplines (eg: nursing, allied health) and more broadly as part of patient-centred care [6–8]. Dignity is central to care in nursing [9, 10], and dignified and respectful care is closely related to patient satisfaction [11, 12].

Article 1 of the Universal Declaration of Human Rights (UDHR) states “all human beings are born free and equal in dignity and rights. They are endowed with reason and consciences and should act towards one another in a spirit of brotherhood” [13]. Professional codes of conduct for healthcare practitioners also advocate for care that protects patients’ dignity. Specifically, the International Council of Nurses’ (ICN) Code of Ethics [14] states ‘inherent in nursing is a respect for human rights, including cultural rights, the right to life and choice, to dignity and to be treated with respect’. The Declaration of Human Rights and the International Council of Nursing Code emphasise the inherent nature of dignity as a fundamental human right.

Despite dignity being entrenched within various codes of conduct, guidelines and standards for patient care, dignity remains a contested concept that is difficult to define, measure and apply to healthcare [15–18]. Indeed, Macklin [16] generated considerable debate among healthcare professionals when she claimed dignity was a useless concept in healthcare, and that it “means no more than respect for persons or their autonomy” (p. 1419) and has “no meaning beyond what is implied by the principle of medical ethics, respect for persons, the need to obtain voluntary, informed consent; the requirement to protect confidentiality; and the need to avoid discrimination and abusive practices” (p. 1419). However, the term ‘dignity’ intersects with other terms such as pride, self-respect, quality of life, wellbeing, hope, self-esteem [5], autonomy, respect, empowerment and communication [19]. Interpretations of human dignity are historically, politically and culturally relative as they are culturally dependent and change over time [20]. Some researchers highlight that it is often easier for practitioners to describe undignified care rather than to articulate dignified care [11, 21]. Additionally, much of the research in relation to dignity focuses on the care of the dying [3, 5, 22] rather than on dignity-preserving personal care [23].

Despite the fact that respect for human dignity is widely promoted and, arguably, lies at the heart of care [24, 25], international reports identify a lack of attention to the dignity of older people in care homes and hospitals [26–29]. According to the findings of the 2018 Care Quality Commission (CQC) NHS Inpatient Survey in the UK, almost one fifth of respondents did not feel they were treated with respect and dignity at all times [27]. In Australia the recent Royal Commission into Aged Care Quality and Safety found that more than half of the complaints made about the quality of care in its residential aged care homes, related to compromises to personal dignity [26]. Hence, there is a needs to focus on how dignity is enacted for people with continence care needs in such settings.

While cognisant of the elusive nature of the term ‘dignity, we propose it could nevertheless be an important value or guiding principle in an ethic of care for older people who need assistance with toileting, incontinence or bladder or bowel care, hereafter referred to as continence care [23, 30]. Continence care has been defined as “the total package tailored to meet the individual needs of patients with bladder and bowel problems” [31]. For the purposes of this paper ‘continence care’ refers to assistance with bladder and/or bowel function which includes supporting a person to maintain continence and manage incontinence. Continence care activities that aim to maintain a person’s continence include helping them to use the toilet or altering the environment to prevent disability incontinence. Continence care activities that centre on managing incontinence include assisting a person to apply or change incontinence products (eg absorbent pads, ostomies and urinary catheters), helping them with their personal hygiene after an episode of incontinence and adopting measures to protect their skin from incontinence-associated dermatitis.

Providing continence care necessarily involves transgressing peoples’ personal space and infringing social norms about privacy and touch [32] and places them at risk of violation of their personal dignity, particularly if they are also care-dependent, cognitively impaired and lack decision-making capacity [33].

High levels of dependence threatens patient dignity, especially with regard to personal care which is at odds with the standards and values that most people embrace throughout their adult lives [33] putting patient dignity at risk. People living permanently in nursing homes are likely to experience urinary and/or faecal incontinence. Urinary incontinence (UI) is defined as ‘the complaint of any involuntary leakage of urine’ [34]. Faecal incontinence (FI) is ‘the involuntary loss of liquid or solid stool that is a social or hygienic problem’ [35]. Incontinence is a widespread condition that ranges in frequency, type and severity. International studies have shown that between 50 and 90% of people living in long-term care experience incontinence and the majority require assistance to maintain continence or manage incontinence [36–40].

Current standards, guidelines, models and principles that are underpinned by autonomy, justice, beneficence, and non-maleficence are largely concerned with issues of consent, competency, and advocacy, but they are silent about how dignity is understood, preserved and enacted on a day-to-day basis, including in the context of providing continence care. Understanding dignity as a concept is critical to understanding incontinence and its management. Previous concept analyses have highlighted key attributes, antecedents and consequences of dignity including privacy, autonomy and respect [41–45]. A previous concept analysis of incontinence and continence undertaken by Dombrowsky & Gray [46] explored some of the key historical descriptions of incontinence highlighting the role that stigma plays. More recently, a concept analysis of UI highlighted the key attributes, the impact of the cultural/environmental context, and the consequences of UI [47]. Whilst these concept analyses of incontinence and dignity provide valuable learning, there is limited research about dignity and incontinence. This has led to a gap in knowledge regarding the requirements for dignified continence care. Understanding the essential attributes of dignity-protective continence care will allow caregivers and healthcare professionals to challenge practices that violate dignity, and recognise opportunities for dignity preservation.

International guidelines and standards about incontinence identify dignity in continence care as an important issue [48–52], however, they do not elaborate on the attributes of dignity-protective continence care. Hence, there is no way to currently quantify if continence care does or does not protect a person’s dignity. Therefore, a systematic analysis of the concept of dignity relating to continence care which explores definitions and understandings of the term in both academic literature and other literature utilised by practitioners and policy makers is timely. The information could be used to design and validate an instrument to measure dignity-protective continence care.

## Aim

The aim of this concept analysis was to explore, describe and explain the concept of dignity as it relates to continence care for older people in long-term care settings.

## Method

Concept analysis is a well-established method that has been used to analyse many concepts in health and social care (for example - human dignity [43], critical health literacy [53], cultural competence [54] and poor care [44]. Concept analyses are integral to examining concepts that are often simply seen as a task or a series of tasks [55, 56]. By using a structured and objective process, researchers can identify critical elements of a given concept, including antecedents, attributes and consequences [55, 57]. Defining the key conceptual elements assists in the development of theory that may be useful in practice, education or further research [58].

The benefit of conducting a concept analysis is that it offers a method to develop a set of defining characteristics that articulates “what counts” as the concept [57]. Specifically, it allows the researcher to (a) formulate a clear, precise theoretical and/or operational definition to be used in the study; (b) choose measurement instruments that accurately reflect the defining characteristics of the concept to be measured; (c) determine if a new instrument is needed (if no extant measure adequately reflects the defining characteristics); and (d) accurately identify the concept when it arises in clinical practice or in qualitative research data [59]. Following the clarification of key concepts, operational definitions can be developed to guide education and clinical practice and to measure the core attributes; in this case, the attributes of dignity-protective continence care.

The goal of this concept analysis was to define ‘dignity in continence care’ based on attributes or “essences” that capture the meaning of the concept under study [56]. There are a number of different concept analysis methods available [see for example, [55, 56, 60–62]. Rodgers [56] highlights that it is the attributes that serve as the true definition of the concept under study and that the definition of the key attributes or the concept is the “primary accomplishment of a concept analysis” [57], p. 91]. In order to define the key concepts, the first four steps of Rodgers [57] evolutionary method of concept analysis were used.

1. Identification and naming of the concept of interest and its surrogate terms,
2. Identification and selection of an appropriate sample for the data collection,
3. Collection of data relevant to identifying attributes and contextual bases of the concept,
4. Analysis of the data to identify key characteristics of the concept.

An inductive processes of analysis which included the definition of the concept of dignity in relation to continence care as well as identifying its alternative statements and terms was used. This approach allowed for the consideration of concepts as dynamic [57] which provided a comprehensive understanding of the concept.

### Sampling

To identify and select an appropriate sample for data collection we used an iterative process that began with identifying key guidance about continence care from contemporary sources and organisations that develop and promote best practice recommendations about incontinence. The websites of several linked organisations were searched for guidance about continence care. Guidance or policy documents were identified from the UK Continence Care Steering Group [48], the UK National Health Service [49], the Royal College of Physicians [50] the Association for Continence Advice [51], Minimum Standards for Continence Care in the UK [52], the Continence Nurses Society of Australia, [63], the Wound, Ostomy and Continence Nurses Society [64], the British Geriatrics Society [65] and the International Consultation on Incontinence (ICI) [66]. As part of this development process, we extracted information that related to dignity in continence care from each of the guidelines. This information was used to support the development of the search strategy. The concepts of interest developed from this process are outlined in Table 1.

**Table 1:** Concepts for search

Concept 1	Concept 2	Concept 3	Concept 4
Dignity	Incontinence	Long-term residential or inpatient formal care and support for day-to-day living in a social or health care setting such as:	Older people
Indignity	Continence	Care homes	Elder
Respect	Bladder incontinence	Nursing homes	Aged
Autonomy	Bowel incontinence	Residential aged care homes	
Privacy	Urinary incontinence	Long-term care	
Empathy	Faecal incontinence	Homes for the aged	
Communication		Aged care homes	
		Assisted living facilities	
Patient centred care			
Person centred care		Inpatient	
Empowerment		Hospital	
Human rights		Palliative care	
		Rehabilitation	

Whilst most of these guidelines state dignity is an important part of care, few provided definitions or examples of what dignity in relation to continence care might look like. Two exceptions to this were found; the first was the British Geriatrics Society [65] campaign (Behind Closed Doors) whose best practice recommendations link dignity to an assessment of the older person’s toileting needs and personal choice and preferences related to continence care. The second was the ICI Guidelines that cite the Dignity in Continence Care Framework developed by Ostaszkiewicz [30].

### Inclusion & exclusion criteria

Inclusion and exclusion criteria were developed based on the initial guidelines and searches. The main requirement was that all studies had to address all of the following four key concepts: four key concepts (i) incontinence or continence, (ii) dignity or indignity, (iii) older people and (iv) facilities or services that provide long-term residential or inpatient formal care and support for day-to-day living (see Table 2). Duplicates were removed except where two or more papers reported different aspects of the same study.

**Table 2:** Inclusion/exclusion criteria

	Inclusion
Concepts of interest	Studies that address all four key concepts (i) incontinence or continence, (ii) dignity or indignity, (iii) older people and (iv) facilities or services that provide long-term residential or inpatient formal care and support for day-to-day living.
Population and setting	Older people (aged 65 or over, or a majority with a mean age of 65) living in care settings that provide long-term residential or inpatient formal care and support for day-to-day living. These care settings are referred to as either care homes, nursing homes, long-term care homes, homes for the aged, aged care homes, assisted living facilities, and residential aged care facilities. In some countries, long-term residential or inpatient formal care is provided in acute or sub-acute care settings such as in hospitals, medical centres, and in rehabilitative or palliative care units.
Study types	Empirical studies (qualitative, quantitative or mixed methods), theoretical papers and reviews of empirical studies (systematic reviews, scoping reviews, integrative reviews or realist reviews) – published in English in any geographic location
Condition(s) or phenomenon of interest	Continence or incontinence. Incontinence could be urinary incontinence or faecal incontinence or both urinary and faecal incontinence. Perceptions and experiences of dignity and continence care
Intervention(s) of interest	Interventions to protect the dignity of older people with continence care needs in care home settings
Year of publication	2009–2019
	**Exclusion**
Concepts of interest	Studies that do not address all four key concepts: (i) incontinence or continence, (ii) dignity or indignity, (iii) older people and (iv) facilities or services that do not provide long-term residential or inpatient formal care and support for day-to-day living. Studies conducted on acute care wards where length of stay is typically brief were excluded.
Population and setting	Studies that relate to people younger than 65 years or conducted in participants’ home, or other community setting or if participants only attended the care home or facility on a daily basis and were not residents or inpatients.
Study types	Publications based on expert opinion, non-peer reviewed papers, non-full text papers including conference abstracts and/or publications in languages other than English
Condition(s) or phenomenon of interest	
Intervention of interest	Medical interventions for the treatment of incontinence

### Search strategy

The relevant surrogate terms extracted from the initial search of the key continence guidelines and existing literature on dignity and continence were incorporated into the search strategy along with recommended terms from the research team. In order to undertake a comprehensive and systematic search, a specialist healthcare librarian assisted with the development of search strategies for each database (MEDLINE Ovid, Embase Ovid, PsycINFO, CINAHL EBSCO, Web of Science, Google Scholar and Cochrane Complete, based on the one developed for MEDLINE (Ovid) (see Table 3). Medical Subject Headings (MeSH) terms were also included to complement the search. Key search terms included truncation of key words, use of thesaurus terms and subject headings, and combining terms and search strings with the appropriate Boolean operators. Date limiters and English language were then applied for each database. The full search strategy, based on the MEDLINE strategy is outlined in Table 3.

**Table 3:** Search strategy developed for MEDLINE

S1	AB (respect OR respected OR respectful* OR autonomy OR privacy OR empathy OR “patient centered care” OR “patient centred care” OR empower* OR “human right*” OR digni* OR personhood) OR TI (respect OR respected OR respectful* OR autonomy OR privacy OR empathy OR “patient centered care” OR “patient centred care” OR empower* OR “human right*” OR digni* OR personhood)
S2	(MH “Personhood”)
S3	(MH “Respect”)
S4	S1 OR S2 OR S3
S5	AB (incontinen* OR continen* OR bladder* OR bowel* OR urinary OR fecal) OR TI (incontinen* OR continen* OR bladder* OR bowel* OR urinary OR fecal)
S6	(MH “Urinary Incontinence”) OR (MH “Fecal Incontinence”)
S7	S5 OR S6
S8	AB (“resident* care” OR “nursing home*” OR hospital* OR inpatient OR “in patient” OR “long term care*” OR “social care” OR “aged care home*” OR “home for the aged”) OR TI (“resident* care” OR “nursing home*” OR hospital* OR inpatient OR “in patient” OR “long term care*” OR “social care” OR “aged care home*” OR “home for the aged”)
S9	(MH “Homes for the Aged”)
S10	(MH “Residential Facilities”) OR (MH “Homes for the Aged”) OR (MH”Assisted Living Facilities”)
S11	(MH “Nursing Homes”)
S12	S8 OR S9 OR S10 OR S11
S13	AB (aged or senior* or “older people” or geriatric*) OR TI (aged or senior* or “older people” or geriatric*)
S14	(MH “Aged+”)
S15	S13 OR S14
S16	S4 AND S7 AND S12 AND S15

### Screening of studies

**Fig. 1:**
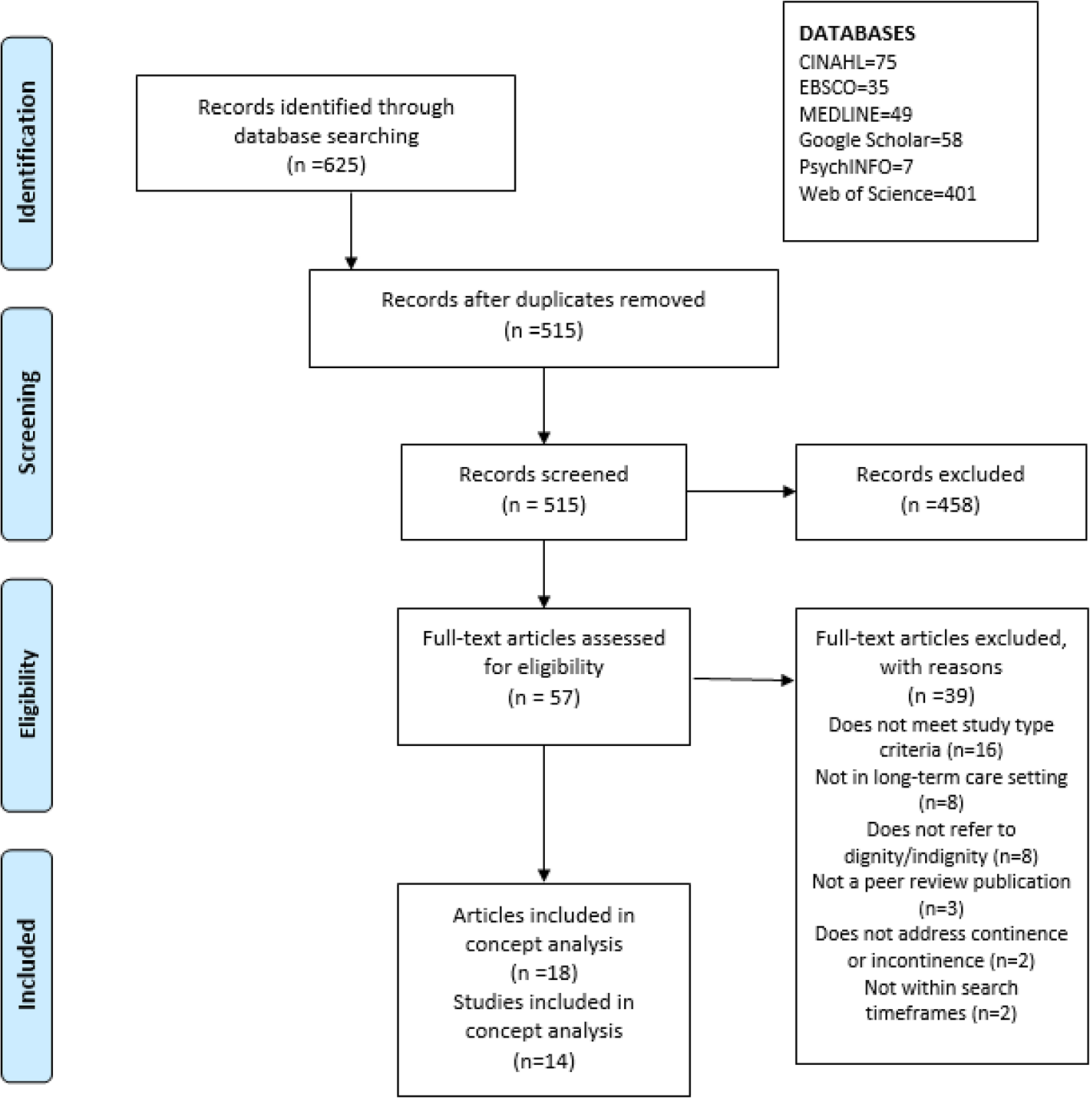
PRISMA Flow Diagram.

All included records were managed in Covidence [67] (systematic review management software) to assist with the review process. All reviewers assessed a sample of at least 25 articles to ensure reliability in application of the inclusion and exclusion criteria. Any discrepancies were resolved via discussion. All records were screened by a minimum of two reviewers. As screening was conducted, conflicts were automatically identified by the Covidence software, and these were then discussed by the review team until consensus was reached. A PRISMA flow chart was used to document all stages of study selection (see Fig. 1).

### Data extraction

As the focus of this review was on the concepts that relate to dignity protective continence care for care-dependent older people, data were extracted with regard to authors, date, sample, country of origin, and key definitions, attributes, contexts and consequences from each of the included records. The following questions were developed to guide the data extraction for the concept analysis.

1. How is dignity defined in continence care for older care-dependent individuals in long-term formal care settings?
2. How is dignity protected in the provision of continence care in long-term formal care settings?
3. What factors play a role in protecting the dignity of care-dependent older individuals in long-term formal care settings in relation to continence care?
4. What are the consequences of not protecting dignity in continence care for care dependent older people, staff and families in long-term formal care settings?

### Data analysis and synthesis

As the purpose of this concept analysis was to explain and describe the concept, common applications and to clarify key attributes, a concept analysis employing Rodgers evolutionary methods was used [56, 57]. In line with this method, we initially explored surrogate terms for ‘dignity’, searching for terms that had similar meanings or that might be related. The next steps in the analysis involved identifying the antecedents of dignity and examining which events or concepts were related to dignity. Undertaking this process allowed us to identify and refine the central attributes of dignity in relation to continence care [55]. Whilst antecedents are factors that must be present before the occurrence of the concept, consequences are events that occur as a result of the concept [44]. At least two researchers read each record, and highlighted specific elements of text that referred to attributes, antecedents or consequences of the concept of dignity in continence care. This inductive approach facilitated data condensation, allowing for comprehensive capture of the key aspects of the concept (dignity-protective continence care). The consequences of undignified continence care were identified for residents and their families, care staff and organisational levels. To further enhance the data analysis process, the key guidelines located in the initial scoping process were examined for references to dignity.

## Results

The bibliographic search resulted in a total of 625 articles from across the relevant databases. After title and abstract screening, and removal of duplicates, 55 articles were reviewed at full-text level. From these, 18 articles [21, 23, 30, 32, 33, 68–80] (see Fig. 1 for the PRISMA flow chart) were included. Among the eligible articles were three studies that were reported in two or more papers. Specifically, the ‘FINCH’ study was reported in three papers [69, 70, 77]. Similarly, Ostaszkiewicz reported on different aspects of the same Grounded theory study about providing continence care in Australian residential aged care homes (RACH) in three papers [32, 72, 73]. Hence, the final number of actual studies was 14. The methods, sample, design, and main findings are described in Supplementary file 1 (Table of Included Studies). Supplementary file 2 (Table of Excluded Studies) includes a list of the 39 studies excluded at full-text level, and the rationale for their exclusion.

Of the 14 eligible studies, five were quantitative, reported in seven papers [21, 69–71, 77, 79, 80], five were qualitative, reported in seven papers [23, 32, 68, 72–75], two were theoretical papers [33, 72] and two were reviews of international research [74, 76]. Five of the studies and one review were from Australia, three studies and one systematic review were from the UK, two studies were conducted in Sweden, one in Canada and one in the USA.

Seven of the included studies offered evidence about dignity from the perspectives of older people with continence care needs. Among these were a review of literature about residents’ quality of life related to UI, their perspectives about being incontinent and care preferences [74], and a realist review to investigate care recipients’ and other stakeholders’ perspectives of what interventions work to reduce and manage FI in care homes in the UK [69, 70, 77]. In addition, we found two qualitative explorative descriptive studies: one of which examined the continence care preferences of people receiving palliative care [78] and the other explored how family relatives of older people in long-term residential aged care understand quality continence care [75]. Evidence was also extracted from a mixed methods study conducted in the UK where older people with UI and/or FI in nursing homes and hospitals were interviewed to explore their perceptions of factors that impact on a person’s dignity in a care setting [21]. The dataset also included: a retrospective cross-sectional study that compared the quality of life of nursing home residents with, and without UI and the effects of UI on residents’ autonomy, mood and dignity in the USA [80] and a cluster randomised controlled trial of the effects of a person-centred approach to continence care on care home residents’ quality of life in Sweden [79].

Four of the included studies offered evidence about dignity from the perspectives of healthcare professionals, care assistants or service providers. Qualitative research on this topic included a study in which 11 Registered Nurses shared their experiences of providing continence care for older people receiving home care, either in their own home or in an assisted living facility in Sweden [68] and another study in which 19 staff from RACH in Australia shared their beliefs and expectations about what constituted the concept of “quality continence care” for aged care residents [23]. We also drew on findings from a Grounded theory study that involved 88 h of field observations in two RACH in Australia, in-depth interviews with 18 nurses and care assistants, and an analysis of 87 accreditation reports about the quality of continence care [32, 72, 73]. Other evidence derived from a mixed methods study to explore dementia care practitioners’ and service managers’ opinions about the challenges they countered in upholding the dignity of older people with dementia in hospitals, care homes and community settings in the UK. The researchers conducted a two-hour workshop to prompt discussion on vignettes about ethically challenging care decisions, including decisions about continence care [71]. Information about dignity-protective continence care was also located in a systematic review of systematic reviews for the management of UI and the promotion of continence with conservative behavioural approaches in older people in care homes [76].

Evidence for the concept analysis was further informed by two theoretical papers that represented a synthesis of findings from prior qualitative research with contemporary biomedical understandings about incontinence with theoretical concepts from the disciplines of nursing, psychology, and sociology [30, 33]. One such paper describes a conceptual model of possible associations between incontinence, care dependence and elder abuse [33]. The other describes a theoretical framework termed ‘The Dignity in Continence Care Framework designed to improve choice, autonomy, and dignity for people who require assistance to maintain continence or manage incontinence [30]. Using a biopsychosocial approach, the framework promotes the dignity of the person as the overarching goal of care; empathic continence care; acknowledgement of personhood; therapeutic communication; authentic partnership in continence care; and acknowledgement of the effects of stigma. Consistent with Rodgers approach [55, 56] for undertaking a concept analysis, the included articles were analysed to identify the key attributes and antecedents of dignity in relation to continence care. Data were also extracted on the consequences of undignified care for patients, carers and organizations.

### Antecedents and key attributes

The initial analysis of the included studies identified 50 different attributes that were then categorised into individual and environmental (physical and social) levels. These individual attributes were then grouped thematically into 6 main domains (respect, empathy, trust, privacy, autonomy and communication). The antecedents and key attributes of dignity in relation to continence care at the individual level are outlined in Table 4.

**Table 4:** Antecedents and attributes of dignity-protective continence care at the individual level

Domain	Attributes	References
Respect	Treating the person as an individual not as an episode of care, i.e. respect for personhood/humanity Ensuring the person’s body is kept clean Adopting a partnership approach that includes listening to and involving family members, carers and the person being cared for in continence care decisions Showing compassion Taking time to address the person’s needs (not rushing care)	[21, 23, 30, 32, 68–73, 75–80]
Empathy	Conveying kindness i.e. offering reassurance, showing tenderness and compassion, Being gentle, i.e. washing with care and using touch appropriately, Acknowledging the impact of stigma	[21, 30, 32, 69, 70, 72–78]
Trust	Establishing a trusting relationship before care happens, Knowing and understanding the person’s biography and pre admission history, understanding the person’s inner experience, Understanding the person’s unique behaviours, Knowing the person’s values and beliefs, Gathering the person’s narrative to develop an individualised continence care plan, Responding to the person’s continence care needs in a timely manner, Ensuring the person feels emotionally and physically safe in continence care interactions	[21, 23, 33, 68–71, 75, 77–79]
Privacy	Closing doors, closing curtains, Ensuring incontinence products remain hidden so they are not visible to others, Concealing the person’s incontinence from others, Being discreet	[21, 23, 32, 68, 70–73, 76–78]
Autonomy	Providing individualised care that includes offering the person a choice and supporting them to make decisions about the gender of carers, toileting preferences and choice of products	[21, 23, 30, 32, 33, 68, 71–75, 78–80]
Communication	Managing one’s emotional responses and body language, Speaking in a calm, soft tone, Picking up on verbal and nonverbal cues Using touch appropriately, Using appropriate language (eg: ‘do you mind’ if as opposed to ‘I must’), Adopting a friendly and gentle attitude, Maintaining a sense of calm and normality about the situation Minimising the socially taboo nature of the problem in the context of the setting Using humour judiciously, Maintaining eye contact	[21, 23, 30, 33, 68–70, 74, 77, 79]

Another key antecedent to dignity-protective continence care related to the environment; the immediate physical and social setting in which the care takes place. There were 15 different attributes that related to the impacts of the environment including the design of the long-term care setting such as ease of access to independent toileting, distance to toilets, obstacles, signage [69, 70, 77] and safety [78]. A further 9 attributes that related to the social environmental were identified. These included; time to deliver care and flexible work practices [32, 69, 70, 72, 73, 77, 79], staff knowledge and beliefs about incontinence [23, 32, 69, 70, 72, 73, 77], an adequate number of staff as well as staff who are trained [23, 30, 32, 33, 69, 70, 72, 73, 77, 79], managerial support and leadership [68–70, 77], a predictable work environment [32, 72, 73] and regulation that does not constrain caring practices [69, 70, 77].

#### Consequences of undignified continence care

The analysis of the included studies identified 23 consequences of undignified continence care that were categorised into 3 levels of impact (resident/family member, staff or organisation). These consequences of undignified continence care, identified from the concept analysis are detailed in Table 5.

**Table 5:** Consequences of undignified continence care

For residents and/or family members	For care staff	For organizations
Vulnerability and threats to social integrity [33] Feeling like a child [21, 74] Feeling like a burden or a nuisance [21] Feeling degraded [78] Feeling unclean/dirty [21, 32, 72, 73] Being embarrassed [21, 23, 68, 74, 75, 78] Feeling ashamed and humiliated, leading to loss of personhood [21, 23, 32, 68, 72, 73, 75] Being anxious, afraid, distressed, agitated [30, 32, 33, 69, 70, 72, 73, 75, 77, 79] Feeling stigmatised [69, 70, 77] Low self-esteem [71, 74] Self-imposed isolation [80] Resistance to care [30, 33, 74] Concerns about odour [78]	Being ethically compromised & morally distressed [33, 71, 72] Feeling stressed, burnt-out, emotionally burdened [30, 33, 79] Feeling devalued in continence care role & subject to low occupational esteem [30, 32, 69] Negative emotions (i.e. disgust, frustration, resentment) [30, 33] Being at risk of internalising stigma [21, 30, 32]	High staff turnover [69, 70, 77, 79] Financial implications [68] Low staff morale [69, 70, 77] Less likely to report undignified care [21] Coercive abuseive or neglectful continence care [33]- Odour of incontinence (if poorly managed) [71]

## Discussion

The purpose of this concept analysis was to identify the essential attributes, antecedents’ and consequences of dignity-protective continence care for older people who are care dependent who require long-term formal care and support for day-to-day living. Our analysis demonstrates that dignified continence care happens when key attributes of care including privacy, respect, autonomy, empathy and trust, communication are the focus of the care encounter. Whilst the concepts identified in the current study are similar to previous concept analyses-including: privacy, autonomy, respect [41–46], stigmna [45] and the impact of the cultural/environmental context on the provision of care [47], prior examinations of dignity as a central concept in the provision of continence care in long-term residential or inpatient formal care settings are lacking.

A key antecedent for dignified care was the development of a therapeutic relationship between the care provider and the care recipient. Sometimes referred to as “person-centred care” this approach is based on a philosophy of care that promotes personal choice and autonomy for people in receipt of care [81–85]. The ability to deliver person-centred care depends in turn on knowing the person [81, 82]. Our analysis of dignity identified six key domains for the provision of dignified continence care including; respect, empathy, trust, privacy, autonomy and communication. These findings align well with the findings of a systematic review by Kogan, Wilber, & Mosqueda [83] who identified six key domains of person-centred care for older adults: (a) holistic or whole-person care, (b) respect and value, (c) choice, (d) dignity, (e) self-determination, and (f) purposeful living or encouragement of continued social roles (p. e1). Whilst there are many similarities between the domains of person-centred care and dignity-protective continence care there are some key differences relating to privacy, empathy and communication.

The findings of this study revealed several practices that could uphold the dignity of older people who are care dependent who require continence care. In practice, nursing home staff engage in several practices that result in residents’ incontinence being contained and thus concealed, with incontinence products, ostensibly to protect residents’ dignity [32]. Whilst the desire to protect residents’ dignity is commendable, the findings of this concept analysis suggest that concealment is only one of several aspects of dignity-protective care.

The use of humour in the care encounter was identified as a key part of therapeutic communication and can be dignity-protective or dignity-violating [86, 87]. Caregivers may use humour judiciously to alleviate fears and protect dignity [86] however they also need to aware of its potential to humiliate people [87].

Dignity-protective continence care is care that is delivered with compassion and respect. Previous studies have highlighted the importance of dignified continence care at end of life [78] however, it is important to consider the subjective nature of dignity that varies by person and throughout life [20]. Receiving continence care may challenge a person’s sense of dignity but for some this may be relatively insignificant compared to other problems (eg: pain or other symptoms) [78]. Dignity may not be as big a concern at the end of life as is it is for a healthy individual [77] and studies suggest that here a loss of dignity may be accepted as a “trade-off” [78]. Matiti and Trorey used the term perceptual adjustment to describe a process by which a person forecasts the potential indignities that he or she expects to suffer whilst in care, “mentally analyses the situation and adjusts to a level that he or she feels comfortable enough to accept” [ [88] p. 741]. In relation to continence care, fears might relate to being ridiculed or humiliated [22, 75], being naked [78], invasion of personal space, loss of self-control [78] and loss of independence [88].

The concept analysis highlighted a number of consequences of undignified continence care for patients, carers and organizations, including shame, humiliation, embarrassment (for patients), stress, burnout and feeling emotionally and ethically challenged (for care staff) and financial and staffing pressures (for organizations). Whilst incontinence itself is not a life threatening illness, it has been viewed by some as a state worse than death [89]. It is difficult to ascertain if it is the incontinence or the care dependence and subsequent loss of dignity that incites fear and dread. We hypothesize that the provision of dignified continence care may mitigate this fear.

Although some international guidelines identified dignity as an important part of care, few attempted to define or give examples of what dignity in relation to continence care might look like. One exception to this is the ‘Dignity in Continence Care Framework’ [30] that promotes the dignity of the person as the overarching goal of care – i.e. dignity as the person perceives it, rather the perspective of the person delivering care. By defining the attributes of dignity protective continence care, our concept analysis builds on, and operationalises the framework. Based on the findings of this analysis, we suggest dignity in continence care (dignity-protective continence care) is operationalised through practices that promote respect, empathy, trust, privacy, autonomy and communication.

Many of the key organizational antecedents to dignified care identified from the analysis suggest that providing dignified continence care requires time and resources. As the population ages, the acuity and complexity of care required in long-term care settings will also increase. Coupled with this, the sector faces severe constraints on budgets, staffing, time and resources, all of which affect the provision of dignified care [26]. By clarifying the concept dignity, and exploring how it is manifested in continence care, the findings of this concept analysis can support reflection on the identified antecedents and attributes as part of appropriate staff education and training [76, 77].

The purpose of this analysis was to develop a better understanding of dignity-protective continence care, i.e. to describe and explain the concept of dignity as it relates to continence care for older people in organisations that provide long-term formal care. Whist literature was retrieved from a variety of sources, using a structured search process, there are some limitations. Studies in languages other than English were not accessed and a significant body of literature may therefore have been excluded. Jacelon [20] makes the point that understandings of dignity are not only historically and politically relative, they are also culturally dependent. The fact that the search yield information from five Western countries is an important limitation.

Moreover, in a seminal article about dignity-conserving care for people at the end of life, Harvey Chochinov pointed out that “If the preservation of dignity is to be a targeted goal of palliation, the patient’s sense of dignity must first be thoroughly understood” [5]. Our search yielded only seven studies about dignity-protective continence care from the perspectives of people with continence care needs. Further research about their experiences and preferences for care is required to deepen our understandings of how to define and thus operationalise dignity-protective continence care. A broader search may have produced a more comprehensive definition of dignity. Additionally, employing an alternative method for the concept analysis may have produced a different outcome (eg Avant & Walker’s).

## Conclusion

This study reveals that although dignity is subjectively experienced, the key attributes of dignity-protective continence care are privacy, respect, autonomy, empathy, trust, and communication, in the caregiving encounter. An understanding of the essential attributes of dignity-protective continence care could allow caregivers and healthcare professionals to challenge practices that violate dignity, and recognize caring opportunities for protecting the dignity of vulnerable and care-dependent older citizens. It could also inform the development of an instrument to evaluate the whether continence care is delivered in a way that protects the person’s dignity.

## Supplementary information

**Additional file 1: Supplementary file 1.** Table of Included Studies.

**Additional file 2: Supplementary file 2.** Table of Excluded Studies.

## Data Availability

All data generated or analysed during this study are included in this published article [and its supplementary information files].

## Abbreviations

ABF: Australian Bladder Foundation
CQC: Care quality commission
FI: Faecal incontinence
UI: Urinary incontinence
ICI: International Consultation on Incontinence
ICN: International Council of Nurses
MeSH: Medical subject headings
NHS: National Health Service
PRISMA: Preferred reporting items for systematic reviews and meta-analyses
RACH: Residential aged care homes
UDHR: Universal Declaration of Human Rights
