# Supplementary material for: A concept analysis of dignity-protective continence care for care dependent older people in long-term care settings
Source: BMC Geriatr. 2020 Jul 29;20:266. doi: 10.1186/s12877-020-01673-x (PMC7392826; doi:10.1186/s12877-020-01673-x)
Supplement: Supplementary file 1 — Additional file 1: Supplementary file 1. Table of Included Studies. [file 12877_2020_1673_MOESM1_ESM.docx]

**Supplementary File 1. Table of Included Studies**

| **Study** | **Design** | **Sample, setting, methods** | **Study findings** | **Attributes (of dignity-protective continence care)** | **Antecedents** *(of dignity-protective continence care)* | **Consequences for staff/organisation** *(of undignified continence care)* | **Consequences for older person/carer**  *(of undignified continence care)* |
| --- | --- | --- | --- | --- | --- | --- | --- |
| ([21](#_ENREF_21)) | A mixed methods study | Phase 1: A review of literature and interviews with 15 nursing home residents & 18 hospital patients.  Phase 2: Observations of practice in nursing homes and hospitals in the UK.  Phase 3: Interviews with 4 residents and patients through workshops and consultation with 2 groups of continence and dignity experts in the UK | Four main themes were identified that were important for dignified care:  (i) communication and relationship, (ii) choice,  (iii) privacy,  (iv) hygiene | Communication,  Choice,  Privacy,  Respect, Acknowledgment of humanity,  Promoting self-esteem | Establishing trust before performing tasks,  Knowing the person,  Ensuring choice re toileting,  Promoting a sense of control,  Ensuring care from people of same gender, Closing doors/curtains during continence care interactions,  Using soft tones when speaking,  Taking time,  Being gentle i.e. washing with care, Making minimal fuss about incontinence,  Being discrete, Providing reassurance, Ensuring the person feels clean,  Responding to verbal and non-verbal cues, Concealing incontinence by using incontinence products | Less likely to report undignified care  Internalised stigma | Vulnerability, Feeling like a child, Embarrassment,  Feeling like a burden,  Feeling like a nuisance,  Being ashamed |
| ([69](#_ENREF_69)) | A qualitative descriptive study | Interviews with 11 Registered Nurses in Sweden working in home care or in an assisted living facility for older people. Inductive analysis resulting in themes | 4 themes: (i) perceptions of  continence care, (ii) an open approach to continence care, (iii) the need for  personalised aid fittings and (iv) the importance of teamwork in continence care. Emphasis was placed on containment among older people—rather than solving the problem. | Communication, Responsiveness,  Trust,  Safety, | Ensuring the person feels clean and comfortable,  Generating a trusting nurse-patient relationship, Providing individualised care,  Working as a team,  Normalising the nature of the care | Continence care not prioritised,  Financial implications | Embarrassment |
| ([70](#_ENREF_70), [71](#_ENREF_71), [78](#_ENREF_78)) | A realist review | A scoping review followed by 5 stakeholder group interviews with care home stakeholders (n=44) followed by theory testing in the UK | 6 broad theory areas identified to explicate how to improve continence care for people with dementia and FI in care homes. Providing intimate and personal care for people with dementia requires particular skills to minimize distress and maximize their comfort. | Empathy,  Privacy,  Respect,  A person-centred approach to care,  Acknowledgement of personhood  A focus on comfort | Knowing the person,  Ensuring a physical environment that optimises continence, Medications,  Ensuring care is not rushed, Communication, Ensuring the person feels clean,  Focussing on comfort,  Good staff leadership,  Adopting a calm manner,  A clinical assessment,  Knowledge of the causes of FI and skills to recognise the person’s preferences,  Dementia-specific continence and toileting skills,  Interventions that allow staff to act on their training | Devaluing of the role staff play, Workforce turnover,  Negative emotions such as disgust,  Low staff morale | Distress,  Internalised stigma,  FI negatively  impacts on dignity, comfort, and quality of life |
| ([72](#_ENREF_72)) | A mixed methods study | A two-hour workshop for dementia care practitioners and service managers in the UK to seek their views about factors related to maintaining dignity in dementia  services through the use of vignettes. | Dementia care Practitioners report experiencing moral decisions and practice constraints when attempting to protect the dignity of people with dementia in clinical and care settings. | Privacy,  Respect | Promoting individual choice,  Knowing the person,  Generating a trusting relationship | Conflict re duty of care,  Presence of a socially taboo odour (if poorly managed) | Potential impacts on pride and self-esteem |
| ([75](#_ENREF_75)) | A descriptive review of literature | A review of qualitative and quantitative research about residents’ quality of life related to urinary incontinence, their perspectives about being incontinent and care preferences. | 10 eligible studies: Residents believe incontinence is inevitable and  intractable. Some attempt to self-manage,  Barriers to residents’ continence or self-management. Low expectations for improved care or continence.  Inadequate information about cognitively impaired residents’ experiences,  however some are distressed when carers’ attempt to provide continence care | Communication Choice  Empathy | Ensuring access to incontinence products such as catheters or pads |  | Feelings of regression to childhood due to dependence on others, Embarrassment,  Low self-esteem |
| ([32](#_ENREF_32), [73](#_ENREF_73), [74](#_ENREF_74)) | A qualitative Grounded theory study | 88 hours of field observations in 2 long-term aged care facilities in Australia, in-depth interviews with 18 nurses and careworkers and an analysis of 87 accreditation reports about the quality of continence care | A basic social problem of ‘caring against the odds’: due to: (i) a highly regulated work environment; (ii) ethically challenging care; (iii) highly dependent residents; and (iv) a devalued role. A basic social process characterised by accommodating strategies, self-protective distancing strategies and reframing care. | Privacy,  Respect, Autonomy, Independence, Self-determination, Acknowledgement of personhood | Ensuring adequate staffing  Being able to predict and manage workload,  Communicating in low tone,  Using touch,  Making eye contact,  Using humour discretionally,  Concealing incontinence from family members, Maintaining privacy,  Containing incontinence with incontinence products  Ensuring the person feels clean | Workforce constraints,  Ethical challenging care,  Internalised stigma  Conflict re duty of care to protect vs promotion of autonomy,  Role devaluation (low occupational status),  Negative emotions such as disgust, | Shame,  Fear,  Humiliation  Family distress and embarrassment  Feeling unclean/dirty |
| ([76](#_ENREF_76)) | A qualitative exploratory descriptive design | Qualitative interviews with 5 next-of-kin family members of residents in an Australian residential aged care home. Data were analysed inductively to build constructions of quality continence care. | Family members valued continence care practices that would maintain residents’ cleanliness and comfort, interpret the behaviours of cognitively impaired residents, optimise personal continence and autonomy, identify and address underlying medical  conditions, and validate family member roles as  advocates. | Compassion,  Empathy,  Autonomy | Ensuring the person feels clean and comfortable, Accommodating the person’s values and beliefs,  Knowing the person’s preferences, Providing timely and regular toileting assistance, Listening to, and involving family members. |  | Humiliation, Feeling dirty,  Embarrassment,  Distress for family members |
| ([23](#_ENREF_23)) | A qualitative exploratory descriptive design | Qualitative interviews with 19 nursing home staff: 8 registered nurses, 4 enrolled nurses, 7 personal care workers working in a nursing home in Australia between 2014–2015. Data were analysed inductively to  identify themes and subthemes. | The key theme was “protecting residents’  dignity” which was supported by the following six subthemes: (i) using pads, ii) providing privacy, (iii) knowing how to “manage” incontinence, (iv) providing timely continence care, (v) considering residents’ continence care preferences and (vi)  communicating sensitively. | Privacy,  Rapport,  Trust | Ensuring the person feels clean and comfortable,  Building report and trust,  Considering the person’s preferences, Knowing the person and their behavioural cues, Adopting communication styles such as hushed tones when doing continence work,  Ensuring adequate staffing levels and resources,  Ensuring care from people of same gender,  Ensuring access to incontinence products |  | Embarrassment,  Shame , Humiliation, |
| ([30](#_ENREF_30)) | A theoretical paper | A synthesis of findings from prior qualitative research with contemporary biomedical understandings about incontinence with theoretical concepts from the disciplines of nursing, psychology, and sociology. | A theoretical framework underpinned by two core concepts: ‘Dignity and Care’ and the promotion of empathic continence care; personhood in dementia; therapeutic communication;  authentic partnership in continence care; acknowledging impact of stigma, social taboos and courtesy  stigma; and the need for a foundational continence assessment | Communication,  Empathy,  Personhood,  Partnership  Stigma | Therapeutic communication, Conveying empathy (i.e. understanding a person’s inner experience), Respect,  Authentic partnership, Acknowledging and rejecting negative effects of stigma and taboos,  Conducting an assessment, Promoting independence, Using humour judiciously | Emotional labour (for staff), Burnout,  Low occupational esteem,  Damaging negative social beliefs that devalues care work | Shame, Humiliation, Denial,  Care resistance, Disengagement |
| ([33](#_ENREF_33)) | A theoretical paper | A synthesis of findings from prior qualitative research with contemporary biomedical understandings about incontinence with theoretical concepts from the disciplines of nursing, psychology, and sociology. | A theoretical model that elucidates possible associations between  incontinence, continence caregiving, and physical and psychological/  emotional abuse, and/or neglect. | Safety,  Support,  Respect for personhood,  Autonomy promoting care  Staff competence | Conveying empathy,  Using skilled communication techniques,  Providing physical and emotional safety,  Being vigilant to pre-empt incontinence  Having dementia-specific with knowledge and skills, including an appreciation of the impact of past trauma on care-recipients’ responses. | Frustration related to unpredictability of incontinence,  Resentment associated with constraints of caregiving,  Negative emotional responses i.e. disgust,  Ethical conflicts about care  Emotional labour | Care resistance,  Distress,  Coercive, abusive or neglectful continence care,  Threats to social integrity  Vulnerability |
| ([77](#_ENREF_77)) | A systematic review of systematic reviews with narrative synthesis. | The PRISMA statement was followed, as were established methods for systematic review of systematic reviews | 5 eligible systematic reviews of high quality were: three specific to intervention studies and two reviewed descriptive studies. Providing older people in care homes with toileting assistance protects their dignity. | Privacy,  Compassion | Providing toileting assistance in a timely manner,  Meeting the person’s essential basic needs |  |  |
| ([79](#_ENREF_79)) | A qualitative descriptive exploratory study | Interviews with 14 patients from tertiary and hospice palliative care units in Canada analysed with a conventional content analysis approach | 3 themes: (i) loss of control, (ii) finding a way to manage, and (iii) caregivers can help and  can hinder. Having to receive continence care was undignifying, however most participants were willing to trade dignity for symptom management such as pain relief.  Patients did not recall being asked about their preferences for bladder and bowel management | Privacy,  Respect, Compassion,  Choice | Having control of mobility,  Having choice, i.e. being asked about care preferences,  Ensuring the person feels clean,  Making the person feel safe, Responding promptly,  Communicating sensitively, Involving family members in care,  Minimising medication side effects,  Ensuring care from people of same gender, |  | Embarrassment (for patient and family),  Feeling degraded, humiliated,  Concerns about odours |
| ([80](#_ENREF_80)) | A cluster randomised controlled prospective cohort  study | 79 residents from 3 residential care facilities in western Sweden: randomised to person-centred continence care (PCCC) or usual continence care. All staff in the intervention group received training in PCCC. | Non-statistically significant increases in the number of assessments and caring actions and an increase in quality of life for the intervention group. | Communication Person centred care, | Adopting a person centred care approach i.e. involving families and carers,  Using skilled communication techniques,  Ensuring education about continence care,  Obtaining resident narratives and integrating them into individualised care planning,  Adopting a partnership model,  Taking time,  Ensuring sufficient organisational resources | Difficulty retaining trained staff,  Staff distress | Distress |
| ([81](#_ENREF_81)) | A retrospective cross-sectional study | Retrospective review of data from the Minimum Data Set and Minnesota Nursing Report Card for 10,683 older nursing home residents in Minnesota, USA. The quality of life questionnaire included questions about, privacy, dignity. | 65.8% had urinary incontinence, a good self-reported quality of life. Although urinary incontinence was not associated with overall quality of life, it decreased the domains of mood, autonomy, and dignity. | Autonomy, Independence | Acknowledging personhood, Accommodating cognitive deficits |  | Embarrassment, Shame, Discomfort,  Self-imposed isolation |
