# Supplementary material for: A concept analysis of dignity-protective continence care for care dependent older people in long-term care settings
Source: BMC Geriatr. 2020 Jul 29;20:266. doi: 10.1186/s12877-020-01673-x (PMC7392826; doi:10.1186/s12877-020-01673-x)
Supplement: Supplementary file 2 — Additional file 2: Supplementary file 2. Table of Excluded Studies. [file 12877_2020_1673_MOESM2_ESM.docx]

**Supplementary File 2. Table of Excluded Studies (n=39)**

| **Authors** | **Exclusion reason** |
| --- | --- |
| 1. Andrews, J. Maintaining continence in people with dementia. Nursing Times. 2013;109(27);20-21 | Does not meet study type criteria |
| 1. Aslan E, Beji NK, Erkan HA, Yalcin O, Gungor F. Urinary incontinence (UI) and quality of life (QoL) of the elderly residing in residential homes in Turkey. Archives of Gerontology and Geriatrics. 2009;49(2):304-310. | Does not refer to dignity / indignity |
| 1. Baillie L, Ford P, Gallagher A, Wainwright P: Nurses’ views on dignity in care. Nursing older people 2009, 21(8). | Not in care setting that provides long-term residential or inpatient formal care and support for day-to-day living |
| 1. Billings, J. (2009). Privacy and Dignity in Continence Care Guidelines. Reflective Guidelines for Health and Social Care Settings. Project report. Canterbury, Centre for Health Services Studies, University of Kent. | Not a peer review publication |
| 1. Billings, J., et al. (2009). Privacy and Dignity in Continence Care Project: Phase 1 Report. Canterbury, Centre for Health Services Studies, University of Kent. | Not a peer review publication |
| 1. Billings, J., et al. (2009). Privacy and Dignity in Continence Care Project: Phase 2 Report. Canterbury, Centre for Health Services Studies, University of Kent | Not a peer review publication |
| 1. Boddington P, Featherstone K: The canary in the coal mine: Continence care for people with dementia in acute hospital wards as a crisis of dehumanization. Bioethics 2018, 32(4):251-260. | Not in care setting that provides long-term residential or inpatient formal care and support for day-to-day living |
| 1. Brady MC, Jamieson K, Bugge C, Hagen S, McClurg D, Chalmers C, Langhorne P. Caring for continence in stroke care settings: a qualitative study of patients' and staff perspectives on the implementation of a new continence care intervention. Clinical Rehabilitation. 2016;30(5):481-494. | Does not refer to dignity / indignity |
| 1. Brown P, Billings J, Wagg A, Potter J: Is it possible to measure what truly matters? the paradox of clinical audit in developing continence service standards for older people. Patient 2010, 3(1):11-23. | Not in care setting that provides long-term residential or inpatient formal care and support for day-to-day living |
| 1. Carlowe J. Older people are not getting discreet and appropriate continence care. British Medical Journal. 2009. 339:b4688 doi: 10.1136/bmj.b4688 | Does not meet study type criteria |
| 1. Drennan VM, Cole L, Iliffe S. A taboo within a stigma? A qualitative study of managing incontinence with people with dementia living at home. BMC Geriatrics, 2011;(11)75. | Not in care setting that provides long-term residential or inpatient formal care and support for day-to-day living |
| 1. Fisher AR: Development of clinical practice guidelines for urinary continence care of adult stroke survivors in acute and rehabilitation settings. Can J Neurosci Nurs. 2014;36(3):16-31. | Not in care setting that provides long-term residential or inpatient formal care and support for day-to-day living |
| 1. Flanagan L, Roe B, Jack B, Barrett J, Chung A, Shaw C, Williams, KS. Systematic review of care intervention studies for the management of incontinence and promotion of continence in older people in care homes with urinary incontinence as the primary focus. Geriatrics & Gerontology International. 2012;12(4):600-611. | Does not refer to dignity / indignity |
| 1. Furlanetto K. & Emond K. “Will I come home incontinent?” A retrospective file review: Incidence of development of incontinence and correlation with length of stay in acute settings for people with dementia or cognitive impairment aged 65 years and over. Collegian. 2016;23(1):79-86. | Not in care setting that provides long-term residential or inpatient formal care and support for day-to-day living |
| 1. Heijkenskjöld KB, Ekstedt M. Lindwall L. The patient’s dignity from the nurse’s perspective. Nursing Ethics. 2010;17(3):313-324. | Does not refer to dignity / indignity |
| 1. Hunter KF. & Wagg AS. Improving nurse engagement in continence care. Nursing: Research and Reviews. 2018;8,1-7. | Does not meet study type criteria |
| 1. Jonasson LL. & Josefsson K. Staff experiences of the management of older adults with urinary incontinence. Healthy Aging Research. 2016;5(16):1-11. | Does not refer to dignity / indignity |
| 1. Kane J. & de Vries K. Dignity in long-term care: An application of Nordenfelt's work. Nursing Ethics. 2017;24(6):744-751. | Does not address continence or incontinence |
| 1. Keegan W. Just put a pad on: Where is the dignity in that? Nursing and Residential Care. 2013;14(3) <https://doi.org/10.12968/nrec.2012.14.3.128> | Does not meet study type criteria |
| 1. Lyons, SS. How Do People Make Continence Care Happen? An Analysis of Organizational Culture in Two Nursing Homes. The Gerontologist. 2009;50(3):327-339. | Does not refer to dignity / indignity |
| 1. McMurdo, M., et al. A cost-effectiveness study of the management of intractable urinary incontinence by urinary catheterization or incontinence pads. J Epidemiol Community Health, 1992;46: 222-226. | Not within search timeframes |
| 1. MacDonald C. & Butler L. Silent no more: elderly women’s stories of living with urinary incontinence in longterm care. J Gerontol Nurs. 2000;33: 14-20. | Not within search timeframes |
| 1. Musa MK, Vinsnes AG, Blekken LE, Harris RG, Goodman C, Boyers D, Norton C. Interventions for treating or managing faecal incontinence in older people living in care homes. [Protocol] Cochrane Database of Systematic Reviews. 2018;11:1465-1858. | Does not refer to dignity / indignity |
| 1. Nazarko L. Dignity in continence care practice. Nursing and Residential Care. 2011;13(9):426-430 | Does not meet study type criteria |
| 1. Nazarko L. Providing dignified continence care to older people at end of life. International Journal of Palliative Nursing. 2019;25(10):504-512. | Does not meet study type criteria |
| 1. Nazarko L. Urinary incontinence: providing respectful, dignified care. British Journal of Community Nursing. 2013;18(2):58-67. | Does not meet study type criteria |
| 1. Nazarko L. Use of continence pads to manage urinary incontinence in older people. British Journal of Community Nursing. 2015;20(8):378-384. | Does not meet study type criteria |
| 1. Noone Y. Continence: A Passing Problem. Australian Ageing Agenda. Jul/Aug. 2010:83-84. | Does not meet study type criteria |
| 1. Oosterveld‐Vlug MG, Pasman HRW, van Gennip IE, Muller MT, Willems DL, Onwuteaka‐Philipsen BD. Dignity and the factors that influence it according to nursing home residents: A qualitative interview study. Journal of Advanced Nursing. 2014;70(1):97-106. | Does not address incontinence or continence care |
| 1. Ostaszkiewicz J. Reframing continence care in care-dependence. Geriatric Nursing. 2017;38(6):520-526. | Does not meet study type criteria |
| 1. Ostaszkiewicz J: A conceptual model of the risk of elder abuse posed by incontinence and care dependence. International Journal of Older People Nursing 2018;13(2):e12182. | Does not meet study type criteria |
| 1. Ostaszkiewicz J, Hutchinson A, Cull E. Cleaning, containing and concealing incontinence in residential aged care facilities: Staff members' constructions of quality continence care. Australian & New Zealand Continence Journal. 2016;22(4). | Does not meet study type criteria |
| 1. Ostaszkiewicz J. New understandings about continence care. Australian Ageing Agenda. Sep/Oct. 2016;50. | Does not meet study type criteria |
| 1. Qian S, Yu P, Hailey DM, Zhang Z, Davy PJ, Nelson MI. Time spent on daytime direct care activities by personal carers in two Australian residential aged care facilities: A time and motion study. Australian Health Review. 2014;38(2):230-237. | Does not refer to dignity / indignity |
| 1. Russell B, Buswell M, Norton C, Malone JR, Harari D, Harwood R, Roe B, Fader M, Drennan VM, Bunn F. Supporting people living with dementia and faecal incontinence. British Journal of Community Nursing. 2017;22(3):110-114. | Does not meet study type criteria |
| 1. Sheridan NF, Kenealy TW, Kidd JD, Schmidt-Busby JIG, Hand JE, Raphael DL, McKillop AM, Rea HH. Patients' engagement in primary care: Powerlessness and compounding jeopardy. A qualitative study. Health Expectations. 2015;18(1):32-43. | Not in a facility or service that provides permanent residential or inpatient care |
| 1. Smith BJ. Faecal incontinence in older people: Delivering effective, dignified care. British Journal of Community Nursing. 2010;15(8):370-374. | Does not meet study type criteria |
| 1. Tauber-Gilmore, M., Addis, G., Zahran, Z., Black, S., Baillie, L., Procter, S., Norton, C. The views of older people and health professionals about dignity in acute hospital care. Journal of Clinical Nursing. 2018;27(1-2):223-234. | Not in care setting that provides long-term residential or inpatient formal care and support for day-to-day living |
| 1. Yates A. Urinary incontinence: promoting independence and dignity. Nursing and Residential Care. 2019(21)3. | Does not meet study type criteria |
